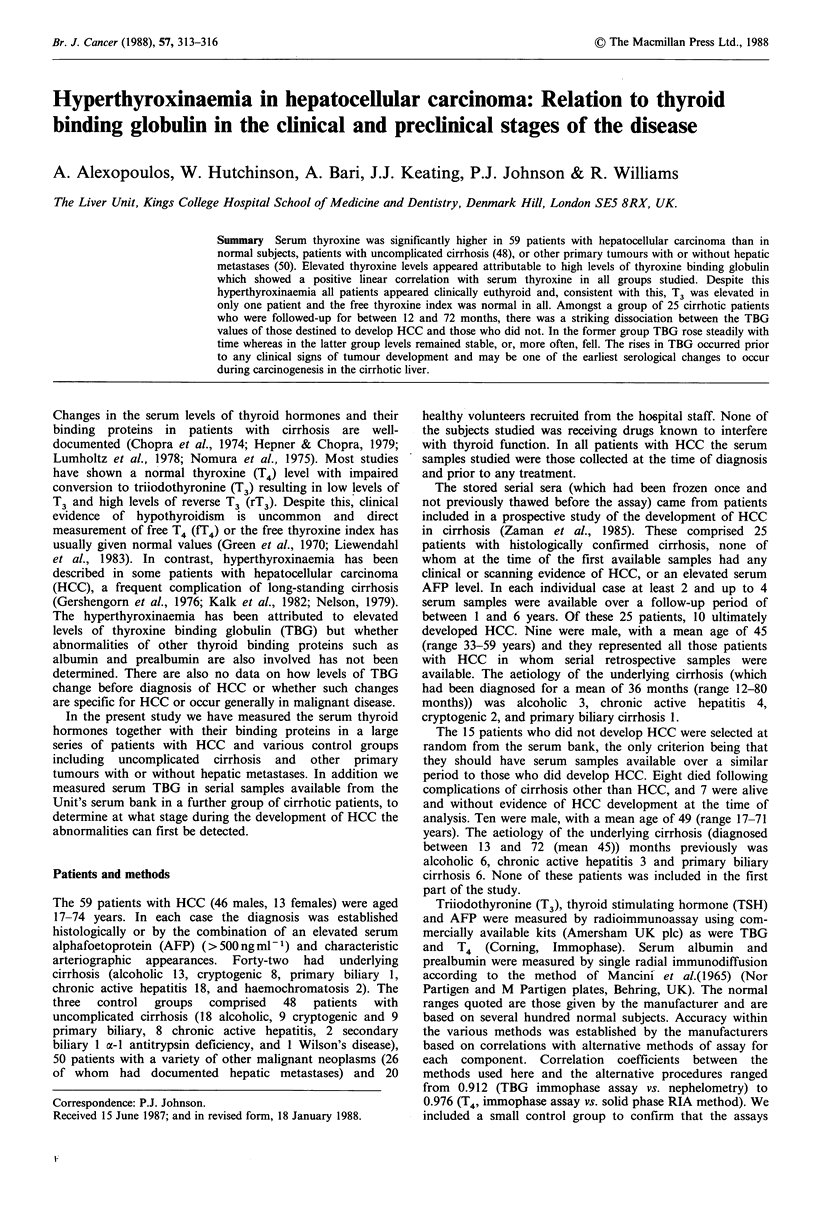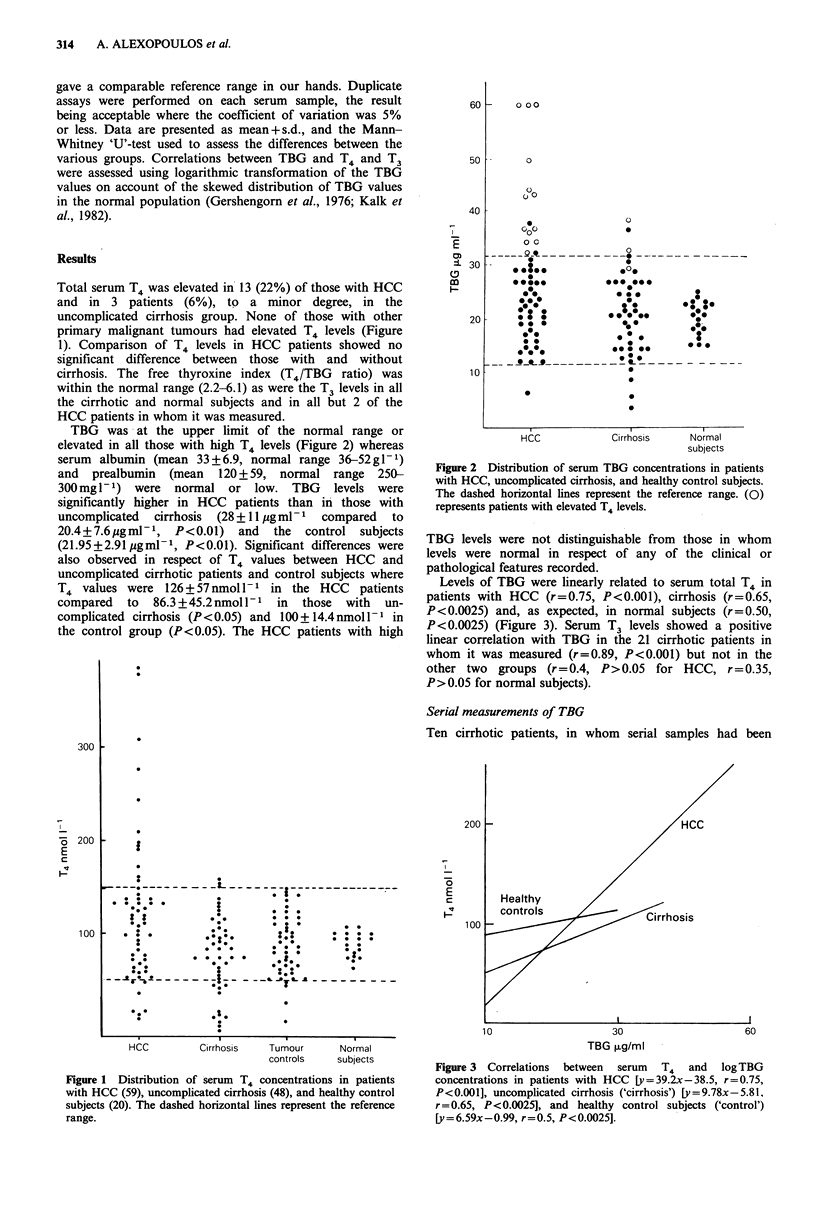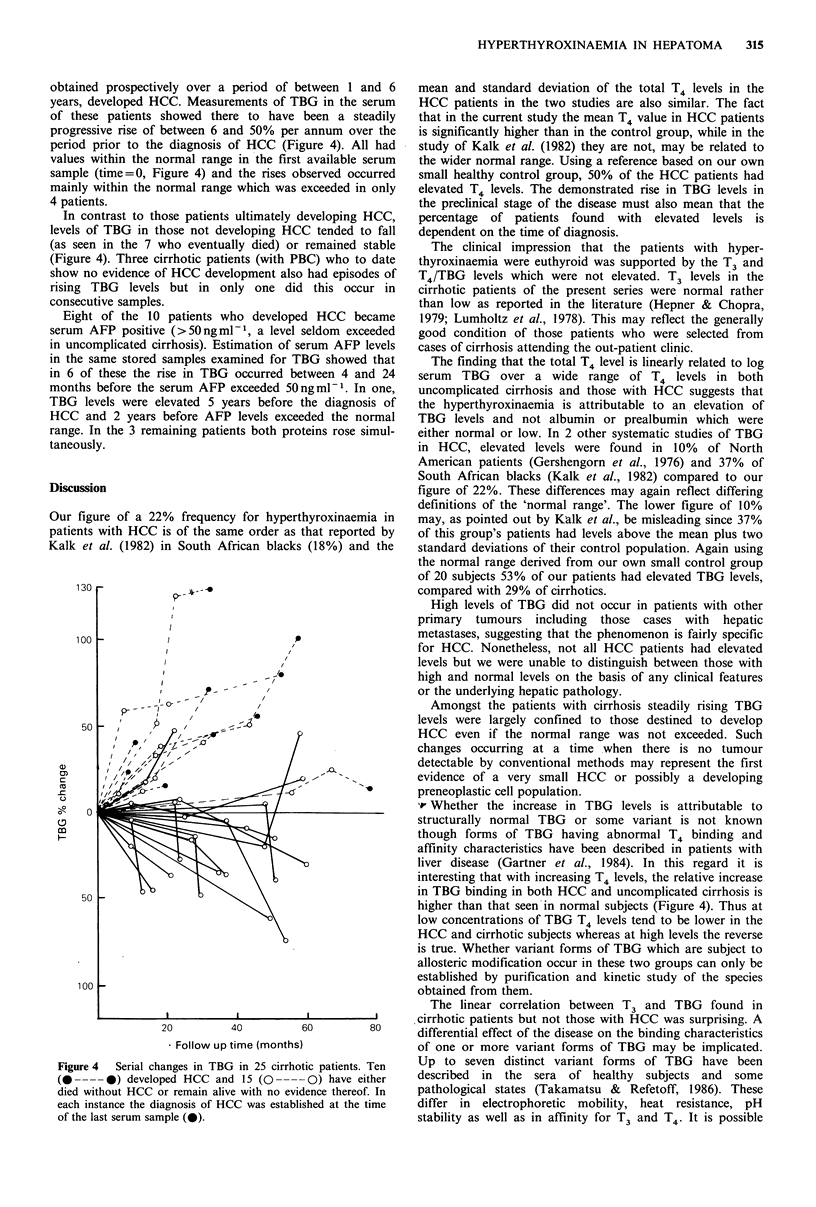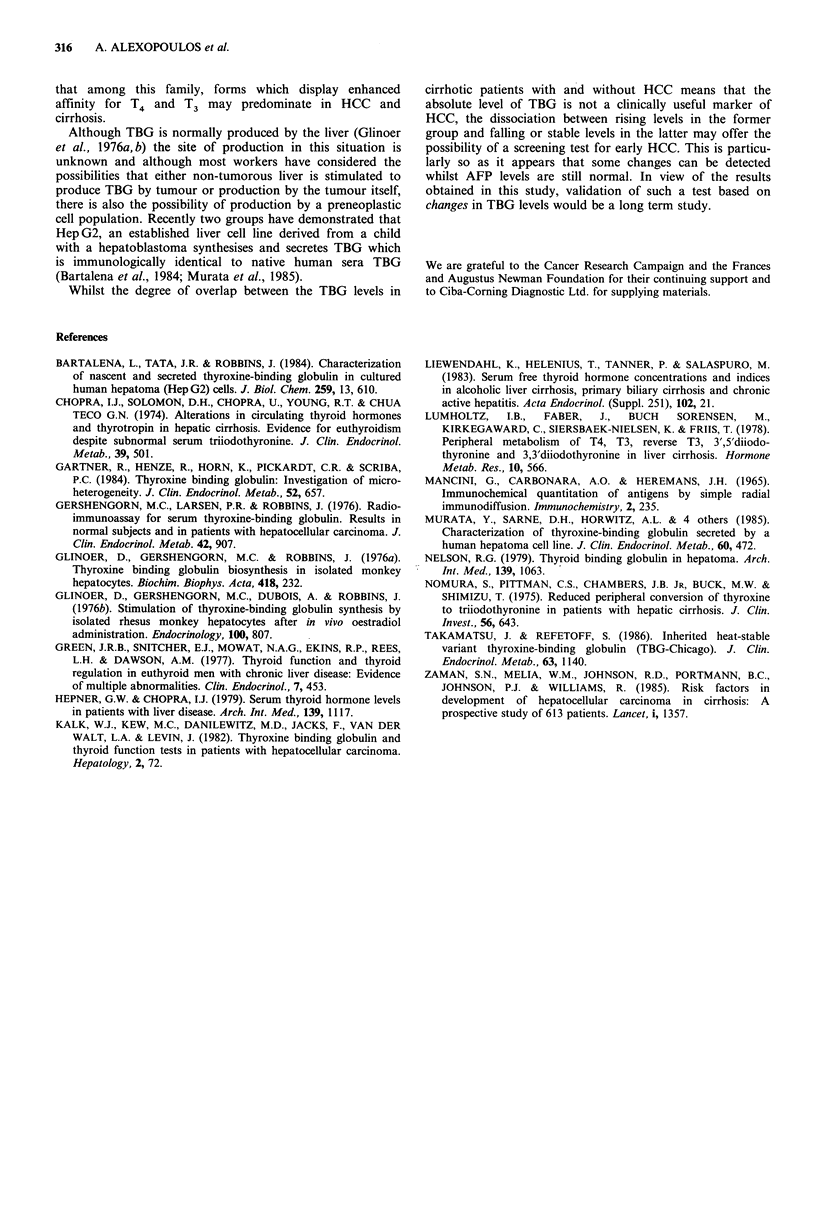# Hyperthyroxinaemia in hepatocellular carcinoma: relation to thyroid binding globulin in the clinical and preclinical stages of the disease.

**DOI:** 10.1038/bjc.1988.69

**Published:** 1988-03

**Authors:** A. Alexopoulos, W. Hutchinson, A. Bari, J. J. Keating, P. J. Johnson, R. Williams

**Affiliations:** Kings College Hospital School of Medicine and Dentistry, Denmark Hill, London, UK.

## Abstract

Serum thyroxine was significantly higher in 59 patients with hepatocellular carcinoma than in normal subjects, patients with uncomplicated cirrhosis (48), or other primary tumours with or without hepatic metastases (50). Elevated thyroxine levels appeared attributable to high levels of thyroxine binding globulin which showed a positive linear correlation with serum thyroxine in all groups studied. Despite this hyperthyroxinaemia all patients appeared clinically euthyroid and, consistent with this, T3 was elevated in only one patient and the free thyroxine index was normal in all. Amongst a group of 25 cirrhotic patients who were followed-up for between 12 and 72 months, there was a striking dissociation between the TBG values of those destined to develop HCC and those who did not. In the former group TBG rose steadily with time whereas in the latter group levels remained stable, or, more often, fell. The rises in TBG occurred prior to any clinical signs of tumour development and may be one of the earliest serological changes to occur during carcinogenesis in the cirrhotic liver.


Br. J. Cancer (1988), 57, 313–316 © The Macmillan Press Ltd., 1988

### Hyperthyroxinaemia in hepatocellular carcinoma: Relation to thyroid binding globulin in the clinical and preclinical stages of the disease

A. Alexopoulos, W. Hutchinson, A. Bari, J.J. Keating, P.J. Johnson & R. Williams

*The Liver Unit, Kings College Hospital School of Medicine and Dentistry, Denmark Hill, London SE5 8RX, UK.*

**Summary** Serum thyroxine was significantly higher in 59 patients with hepatocellular carcinoma than in normal subjects, patients with uncomplicated cirrhosis (48), or other primary tumours with or without hepatic metastases (50). Elevated thyroxine levels appeared attributable to high levels of thyroxine binding globulin which showed a positive linear correlation with serum thyroxine in all groups studied. Despite this hyperthyroxinaemia all patients appeared clinically euthyroid and, consistent with this, $T_3$ was elevated in only one patient and the free thyroxine index was normal in all. Amongst a group of 25 cirrhotic patients who were followed-up for between 12 and 72 months, there was a striking dissociation between the TBG values of those destined to develop HCC and those who did not. In the former group TBG rose steadily with time whereas in the latter group levels remained stable, or, more often, fell. The rises in TBG occurred prior to any clinical signs of tumour development and may be one of the earliest serological changes to occur during carcinogenesis in the cirrhotic liver.

Changes in the serum levels of thyroid hormones and their binding proteins in patients with cirrhosis are well-documented (Chopra *et al.*, 1974; Hepner & Chopra, 1979; Lumholtz *et al.*, 1978; Nomura *et al.*, 1975). Most studies have shown a normal thyroxine ($T_4$) level with impaired conversion to triiodothyronine ($T_3$) resulting in low levels of $T_3$ and high levels of reverse $T_3$ ($rT_3$). Despite this, clinical evidence of hypothyroidism is uncommon and direct measurement of free $T_4$ ($fT_4$) or the free thyroxine index has usually given normal values (Green *et al.*, 1970; Liewendahl *et al.*, 1983). In contrast, hyperthyroxinaemia has been described in some patients with hepatocellular carcinoma (HCC), a frequent complication of long-standing cirrhosis (Gershengorn *et al.*, 1976; Kalk *et al.*, 1982; Nelson, 1979). The hyperthyroxinaemia has been attributed to elevated levels of thyroxine binding globulin (TBG) but whether abnormalities of other thyroid binding proteins such as albumin and prealbumin are also involved has not been determined. There are also no data on how levels of TBG change before diagnosis of HCC or whether such changes are specific for HCC or occur generally in malignant disease.

In the present study we have measured the serum thyroid hormones together with their binding proteins in a large series of patients with HCC and various control groups including uncomplicated cirrhosis and other primary tumours with or without hepatic metastases. In addition we measured serum TBG in serial samples available from the Unit's serum bank in a further group of cirrhotic patients, to determine at what stage during the development of HCC the abnormalities can first be detected.

### Patients and methods

The 59 patients with HCC (46 males, 13 females) were aged 17–74 years. In each case the diagnosis was established histologically or by the combination of an elevated serum alphafoetoprotein (AFP) ($>500\,\text{ng ml}^{-1}$) and characteristic arteriographic appearances. Forty-two had underlying cirrhosis (alcoholic 13, cryptogenic 8, primary biliary 1, chronic active hepatitis 18, and haemochromatosis 2). The three control groups comprised 48 patients with uncomplicated cirrhosis (18 alcoholic, 9 cryptogenic and 9 primary biliary, 8 chronic active hepatitis, 2 secondary biliary 1 $\alpha$-1 antitrypsin deficiency, and 1 Wilson's disease), 50 patients with a variety of other malignant neoplasms (26 of whom had documented hepatic metastases) and 20 healthy volunteers recruited from the hospital staff. None of the subjects studied was receiving drugs known to interfere with thyroid function. In all patients with HCC the serum samples studied were those collected at the time of diagnosis and prior to any treatment.

The stored serial sera (which had been frozen once and not previously thawed before the assay) came from patients included in a prospective study of the development of HCC in cirrhosis (Zaman *et al.*, 1985). These comprised 25 patients with histologically confirmed cirrhosis, none of whom at the time of the first available samples had any clinical or scanning evidence of HCC, or an elevated serum AFP level. In each individual case at least 2 and up to 4 serum samples were available over a follow-up period of between 1 and 6 years. Of these 25 patients, 10 ultimately developed HCC. Nine were male, with a mean age of 45 (range 33–59 years) and they represented all those patients with HCC in whom serial retrospective samples were available. The aetiology of the underlying cirrhosis (which had been diagnosed for a mean of 36 months (range 12–80 months)) was alcoholic 3, chronic active hepatitis 4, cryptogenic 2, and primary biliary cirrhosis 1.

The 15 patients who did not develop HCC were selected at random from the serum bank, the only criterion being that they should have serum samples available over a similar period to those who did develop HCC. Eight died following complications of cirrhosis other than HCC, and 7 were alive and without evidence of HCC development at the time of analysis. Ten were male, with a mean age of 49 (range 17–71 years). The aetiology of the underlying cirrhosis (diagnosed between 13 and 72 (mean 45)) months previously was alcoholic 6, chronic active hepatitis 3 and primary biliary cirrhosis 6. None of these patients was included in the first part of the study.

Triiodothyronine ($T_3$), thyroid stimulating hormone (TSH) and AFP were measured by radioimmunoassay using commercially available kits (Amersham UK plc) as were TBG and $T_4$ (Corning, Immophase). Serum albumin and prealbumin were measured by single radial immunodiffusion according to the method of Mancini *et al.*(1965) (Nor Partigen and M Partigen plates, Behring, UK). The normal ranges quoted are those given by the manufacturer and are based on several hundred normal subjects. Accuracy within the various methods was established by the manufacturers based on correlations with alternative methods of assay for each component. Correlation coefficients between the methods used here and the alternative procedures ranged from 0.912 (TBG immophase assay *vs.* nephelometry) to 0.976 ($T_4$, immophase assay *vs.* solid phase RIA method). We included a small control group to confirm that the assays

Correspondence: P.J. Johnson.
Received 15 June 1987; and in revised form, 18 January 1988.

F

gave a comparable reference range in our hands. Duplicate assays were performed on each serum sample, the result being acceptable where the coefficient of variation was 5% or less. Data are presented as mean+s.d., and the Mann–Whitney 'U'-test used to assess the differences between the various groups. Correlations between TBG and $T_4$ and $T_3$ were assessed using logarithmic transformation of the TBG values on account of the skewed distribution of TBG values in the normal population (Gershengorn et al., 1976; Kalk et al., 1982).

### Results

Total serum $T_4$ was elevated in 13 (22%) of those with HCC and in 3 patients (6%), to a minor degree, in the uncomplicated cirrhosis group. None of those with other primary malignant tumours had elevated $T_4$ levels (Figure 1). Comparison of $T_4$ levels in HCC patients showed no significant difference between those with and without cirrhosis. The free thyroxine index ($T_4$/TBG ratio) was within the normal range (2.2–6.1) as were the $T_3$ levels in all the cirrhotic and normal subjects and in all but 2 of the HCC patients in whom it was measured.

TBG was at the upper limit of the normal range or elevated in all those with high $T_4$ levels (Figure 2) whereas serum albumin (mean $33 \pm 6.9$, normal range 36–52 g l$^{-1}$) and prealbumin (mean $120 \pm 59$, normal range 250–300 mg l$^{-1}$) were normal or low. TBG levels were significantly higher in HCC patients than in those with uncomplicated cirrhosis ($28 \pm 11\,\mu g\,ml^{-1}$ compared to $20.4 \pm 7.6\,\mu g\,ml^{-1}$, $P<0.01$) and the control subjects ($21.95 \pm 2.91\,\mu g\,ml^{-1}$, $P<0.01$). Significant differences were also observed in respect of $T_4$ values between HCC and uncomplicated cirrhotic patients and control subjects where $T_4$ values were $126 \pm 57\,nmol\,l^{-1}$ in the HCC patients compared to $86.3 \pm 45.2\,nmol\,l^{-1}$ in those with uncomplicated cirrhosis ($P<0.05$) and $100 \pm 14.4\,nmol\,l^{-1}$ in the control group ($P<0.05$). The HCC patients with high

**Figure 1** Distribution of serum $T_4$ concentrations in patients with HCC (59), uncomplicated cirrhosis (48), and healthy control subjects (20). The dashed horizontal lines represent the reference range.

**Figure 2** Distribution of serum TBG concentrations in patients with HCC, uncomplicated cirrhosis, and healthy control subjects. The dashed horizontal lines represent the reference range. (○) represents patients with elevated $T_4$ levels.

TBG levels were not distinguishable from those in whom levels were normal in respect of any of the clinical or pathological features recorded.

Levels of TBG were linearly related to serum total $T_4$ in patients with HCC ($r=0.75$, $P<0.001$), cirrhosis ($r=0.65$, $P<0.0025$) and, as expected, in normal subjects ($r=0.50$, $P<0.0025$) (Figure 3). Serum $T_3$ levels showed a positive linear correlation with TBG in the 21 cirrhotic patients in whom it was measured ($r=0.89$, $P<0.001$) but not in the other two groups ($r=0.4$, $P>0.05$ for HCC, $r=0.35$, $P>0.05$ for normal subjects).

### *Serial measurements of TBG*

Ten cirrhotic patients, in whom serial samples had been

**Figure 3** Correlations between serum $T_4$ and log TBG concentrations in patients with HCC [$y=39.2x-38.5$, $r=0.75$, $P<0.001$], uncomplicated cirrhosis ('cirrhosis') [$y=9.78x-5.81$, $r=0.65$, $P<0.0025$], and healthy control subjects ('control') [$y=6.59x-0.99$, $r=0.5$, $P<0.0025$].

obtained prospectively over a period of between 1 and 6 years, developed HCC. Measurements of TBG in the serum of these patients showed there to have been a steadily progressive rise of between 6 and 50% per annum over the period prior to the diagnosis of HCC (Figure 4). All had values within the normal range in the first available serum sample (time = 0, Figure 4) and the rises observed occurred mainly within the normal range which was exceeded in only 4 patients.

In contrast to those patients ultimately developing HCC, levels of TBG in those not developing HCC tended to fall (as seen in the 7 who eventually died) or remained stable (Figure 4). Three cirrhotic patients (with PBC) who to date show no evidence of HCC development also had episodes of rising TBG levels but in only one did this occur in consecutive samples.

Eight of the 10 patients who developed HCC became serum AFP positive ($>50\,ng\,ml^{-1}$, a level seldom exceeded in uncomplicated cirrhosis). Estimation of serum AFP levels in the same stored samples examined for TBG showed that in 6 of these the rise in TBG occurred between 4 and 24 months before the serum AFP exceeded $50\,ng\,ml^{-1}$. In one, TBG levels were elevated 5 years before the diagnosis of HCC and 2 years before AFP levels exceeded the normal range. In the 3 remaining patients both proteins rose simultaneously.

### Discussion

Our figure of a 22% frequency for hyperthyroxinaemia in patients with HCC is of the same order as that reported by Kalk *et al.* (1982) in South African blacks (18%) and the

**Figure 4** Serial changes in TBG in 25 cirrhotic patients. Ten (●----●) developed HCC and 15 (○----○) have either died without HCC or remain alive with no evidence thereof. In each instance the diagnosis of HCC was established at the time of the last serum sample (●).

mean and standard deviation of the total $T_4$ levels in the HCC patients in the two studies are also similar. The fact that in the current study the mean $T_4$ value in HCC patients is significantly higher than in the control group, while in the study of Kalk *et al.* (1982) they are not, may be related to the wider normal range. Using a reference based on our own small healthy control group, 50% of the HCC patients had elevated $T_4$ levels. The demonstrated rise in TBG levels in the preclinical stage of the disease must also mean that the percentage of patients found with elevated levels is dependent on the time of diagnosis.

The clinical impression that the patients with hyperthyroxinaemia were euthyroid was supported by the $T_3$ and $T_4$/TBG levels which were not elevated. $T_3$ levels in the cirrhotic patients of the present series were normal rather than low as reported in the literature (Hepner & Chopra, 1979; Lumholtz *et al.*, 1978). This may reflect the generally good condition of those patients who were selected from cases of cirrhosis attending the out-patient clinic.

The finding that the total $T_4$ level is linearly related to log serum TBG over a wide range of $T_4$ levels in both uncomplicated cirrhosis and those with HCC suggests that the hyperthyroxinaemia is attributable to an elevation of TBG levels and not albumin or prealbumin which were either normal or low. In 2 other systematic studies of TBG in HCC, elevated levels were found in 10% of North American patients (Gershengorn *et al.*, 1976) and 37% of South African blacks (Kalk *et al.*, 1982) compared to our figure of 22%. These differences may again reflect differing definitions of the 'normal range'. The lower figure of 10% may, as pointed out by Kalk *et al.*, be misleading since 37% of this group's patients had levels above the mean plus two standard deviations of their control population. Again using the normal range derived from our own small control group of 20 subjects 53% of our patients had elevated TBG levels, compared with 29% of cirrhotics.

High levels of TBG did not occur in patients with other primary tumours including those cases with hepatic metastases, suggesting that the phenomenon is fairly specific for HCC. Nonetheless, not all HCC patients had elevated levels but we were unable to distinguish between those with high and normal levels on the basis of any clinical features or the underlying hepatic pathology.

Amongst the patients with cirrhosis steadily rising TBG levels were largely confined to those destined to develop HCC even if the normal range was not exceeded. Such changes occurring at a time when there is no tumour detectable by conventional methods may represent the first evidence of a very small HCC or possibly a developing preneoplastic cell population.

Whether the increase in TBG levels is attributable to structurally normal TBG or some variant is not known though forms of TBG having abnormal $T_4$ binding and affinity characteristics have been described in patients with liver disease (Gartner *et al.*, 1984). In this regard it is interesting that with increasing $T_4$ levels, the relative increase in TBG binding in both HCC and uncomplicated cirrhosis is higher than that seen in normal subjects (Figure 4). Thus at low concentrations of TBG $T_4$ levels tend to be lower in the HCC and cirrhotic subjects whereas at high levels the reverse is true. Whether variant forms of TBG which are subject to allosteric modification occur in these two groups can only be established by purification and kinetic study of the species obtained from them.

The linear correlation between $T_3$ and TBG found in cirrhotic patients but not those with HCC was surprising. A differential effect of the disease on the binding characteristics of one or more variant forms of TBG may be implicated. Up to seven distinct variant forms of TBG have been described in the sera of healthy subjects and some pathological states (Takamatsu & Refetoff, 1986). These differ in electrophoretic mobility, heat resistance, pH stability as well as in affinity for $T_3$ and $T_4$. It is possible that among this family, forms which display enhanced affinity for $T_4$ and $T_3$ may predominate in HCC and cirrhosis.

Although TBG is normally produced by the liver (Glinoer *et al.*, 1976*a*, *b*) the site of production in this situation is unknown and although most workers have considered the possibilities that either non-tumorous liver is stimulated to produce TBG by tumour or production by the tumour itself, there is also the possibility of production by a preneoplastic cell population. Recently two groups have demonstrated that Hep G2, an established liver cell line derived from a child with a hepatoblastoma synthesises and secretes TBG which is immunologically identical to native human sera TBG (Bartalena *et al.*, 1984; Murata *et al.*, 1985).

Whilst the degree of overlap between the TBG levels in cirrhotic patients with and without HCC means that the absolute level of TBG is not a clinically useful marker of HCC, the dissociation between rising levels in the former group and falling or stable levels in the latter may offer the possibility of a screening test for early HCC. This is particularly so as it appears that some changes can be detected whilst AFP levels are still normal. In view of the results obtained in this study, validation of such a test based on *changes* in TBG levels would be a long term study.

We are grateful to the Cancer Research Campaign and the Frances and Augustus Newman Foundation for their continuing support and to Ciba-Corning Diagnostic Ltd. for supplying materials.

### References

BARTALENA, L., TATA, J.R. & ROBBINS, J. (1984). Characterization of nascent and secreted thyroxine-binding globulin in cultured human hepatoma (Hep G2) cells. *J. Biol. Chem.* **259**, 13, 610.

CHOPRA, I.J., SOLOMON, D.H., CHOPRA, U., YOUNG, R.T. & CHUA TECO G.N. (1974). Alterations in circulating thyroid hormones and thyrotropin in hepatic cirrhosis. Evidence for euthyroidism despite subnormal serum triiodothyronine. *J. Clin. Endocrinol. Metab.*, **39**, 501.

GARTNER, R., HENZE, R., HORN, K., PICKARDT, C.R. & SCRIBA, P.C. (1984). Thyroxine binding globulin: Investigation of microheterogeneity. *J. Clin. Endocrinol. Metab.*, **52**, 657.

GERSHENGORN, M.C., LARSEN, P.R. & ROBBINS, J. (1976). Radioimmunoassay for serum thyroxine-binding globulin. Results in normal subjects and in patients with hepatocellular carcinoma. *J. Clin. Endocrinol. Metab.* **42**, 907.

GLINOER, D., GERSHENGORN, M.C. & ROBBINS, J. (1976*a*). Thyroxine binding globulin biosynthesis in isolated monkey hepatocytes. *Biochim. Biophys. Acta*, **418**, 232.

GLINOER, D., GERSHENGORN, M.C., DUBOIS, A. & ROBBINS, J. (1976*b*). Stimulation of thyroxine-binding globulin synthesis by isolated rhesus monkey hepatocytes after *in vivo* oestradiol administration. *Endocrinology*, **100**, 807.

GREEN, J.R.B., SNITCHER, E.J., MOWAT, N.A.G., EKINS, R.P., REES, L.H. & DAWSON, A.M. (1977). Thyroid function and thyroid regulation in euthyroid men with chronic liver disease: Evidence of multiple abnormalities. *Clin. Endocrinol.*, **7**, 453.

HEPNER, G.W. & CHOPRA, I.J. (1979). Serum thyroid hormone levels in patients with liver disease. *Arch. Int. Med.*, **139**, 1117.

KALK, W.J., KEW, M.C., DANILEWITZ, M.D., JACKS, F., VAN DER WALT, L.A. & LEVIN, J. (1982). Thyroxine binding globulin and thyroid function tests in patients with hepatocellular carcinoma. *Hepatology*, **2**, 72.

LIEWENDAHL, K., HELENIUS, T., TANNER, P. & SALASPURO, M. (1983). Serum free thyroid hormone concentrations and indices in alcoholic liver cirrhosis, primary biliary cirrhosis and chronic active hepatitis. *Acta Endocrinol.* (Suppl. 251), **102**, 21.

LUMHOLTZ, I.B., FABER, J., BUCH SORENSEN, M., KIRKEGAWARD, C., SIERSBAEK-NIELSEN, K. & FRIIS, T. (1978). Peripheral metabolism of T4, T3, reverse T3, 3′,5′diiodothyronine and 3,3′diiodothyronine in liver cirrhosis. *Hormone Metab. Res.*, **10**, 566.

MANCINI, G., CARBONARA, A.O. & HEREMANS, J.H. (1965). Immunochemical quantitation of antigens by simple radial immunodiffusion. *Immunochemistry*, **2**, 235.

MURATA, Y., SARNE, D.H., HORWITZ, A.L. & 4 others (1985). Characterization of thyroxine-binding globulin secreted by a human hepatoma cell line. *J. Clin. Endocrinol. Metab.*, **60**, 472.

NELSON, R.G. (1979). Thyroid binding globulin in hepatoma. *Arch. Int. Med.*, **139**, 1063.

NOMURA, S., PITTMAN, C.S., CHAMBERS, J.B. JR, BUCK, M.W. & SHIMIZU, T. (1975). Reduced peripheral conversion of thyroxine to triiodothyronine in patients with hepatic cirrhosis. *J. Clin. Invest.*, **56**, 643.

TAKAMATSU, J. & REFETOFF, S. (1986). Inherited heat-stable variant thyroxine-binding globulin (TBG-Chicago). *J. Clin. Endocrinol. Metab.*, **63**, 1140.

ZAMAN, S.N., MELIA, W.M., JOHNSON, R.D., PORTMANN, B.C., JOHNSON, P.J. & WILLIAMS, R. (1985). Risk factors in development of hepatocellular carcinoma in cirrhosis: A prospective study of 613 patients. *Lancet*, **i**, 1357.